# Predicting diagnostic conversion from mild cognitive impairment to Alzheimer's disease: A Bayesian hierarchical model approach using ADNI patient data

**DOI:** 10.1177/13872877251360228

**Published:** 2025-07-20

**Authors:** Hugo Senra, Maria Conceição Costa, Isabel Pereira, Daniel Agostinho, Miguel Castelo-Branco, for the Alzheimer’s Disease Neuroimaging Initiative

**Affiliations:** 1 Institute of Electronics and Informatics Engineering of Aveiro (IEETA), University of Aveiro, Aveiro, Portugal; 2 School of Health and Social Care, University of Essex, Colchester, UK; 3 Department of Mathematics, University of Aveiro, Aveiro, Portugal; 4 Center for Research & Development in Mathematics and Applications (CIDMA), University of Aveiro, Aveiro, Portugal; 5 Coimbra Institute for Biomedical Imaging and Translational Research (CIBIT), ICNAS, Faculty of Medicine, University of Coimbra, Coimbra, Portugal; 6 Center for Informatics and Systems (CISUC), University of Coimbra, Coimbra, Portugal

**Keywords:** ADNI data, Alzheimer's disease, Bayesian hierarchical models‌, cognitive assessment, diagnostic conversion

## Abstract

**Background:**

There is still need for a better understanding of which specific follow-up medical assessments might offer greater predictive value for diagnostic conversion from mild cognitive impairment (MCI) to Alzheimer's disease (AD).

**Objective:**

To examine the longitudinal predictive importance of follow-up medical assessments to detect diagnostic conversion from MCI to AD.

**Methods:**

A sample of 572 participants from the ADNI database with valid data at baseline medical visit were included. Bayesian hierarchical models were employed to investigate longitudinal predictors of diagnostic conversion in a 36-month medical follow-up cohort, for measures of cognitive function, psychopathological symptoms, and demographical data. An additional 48-month medical follow-up cohort was considered to investigate the predictive importance of cerebrospinal fluid biomarkers (Aβ_42_/Aβ_40_ ratio) for diagnostic conversion.

**Results:**

Mini-mental State Examination (MMSE) (β = −2.6; 95% HDI: [−3.6–−1.5]) and Clinical Dementia Rating scale Sum of Boxes (CDR-SB) (β = 5.6; 95% HDI: [4.3–7.0]) can predict diagnostic conversion from MCI to AD over a 36-month medical follow-up, with CDR-SB showing the greatest predictive importance in all Bayesian models. Higher scores on CDR-SB were associated with increased risk for a diagnosis conversion, approximately 30% greater probability at 24-month follow-up, and > 50% greater probability at 36-month follow-up.

**Conclusions:**

The CDR-SB provides a reliable cognitive assessment to detect diagnostic conversion from MCI to AD over a period of 36 months, which is key to help clinicians screening for early diagnosis of AD using affordable non-invasive procedures.

## Introduction

Current evidence suggests mild cognitive impairment (MCI) in middle aged and older adults to be a potential prodromal stage of dementia, meaning that many individuals with MCI might see at some point their diagnosis being converted to Alzheimer's disease (AD).^1–4^ Medical and cognitive follow-up assessment of individuals with MCI is therefore key for anticipating and managing early stages of dementia, particularly AD.^1–3^ The typical follow-up assessment protocol for adult MCI patients includes cognitive function (neuropsychological assessment), brain structures assessment using neuroimaging, cerebrospinal fluid (CSF) biomarkers, and psychopathological symptoms.Click or tap here to enter text.^5,6^ The information given by these assessments is clinically relevant, but there is still need for a better understanding of which specific follow-up assessments might offer greater predictive value for diagnostic conversion from MCI to AD.^4,7^

Recent literature has drawn attention to biomarkers of diagnostic conversion from MCI to AD coming from structural neuroimaging measures (e.g., magnetic resonance imaging (MRI)). Reduced cortical volumes and thickness have been soundly associated with greater risk for further AD.^8–11^ In addition, frequent moderate to severe MRI-visible perivascular spaces in the centrum semiovale were found to significantly predict diagnostic conversion from MCI to AD (hazard ratios ranging from 2.0 to 2.7).^12^ Shape diffeomorphometry patterns of subcortical and ventricular structures were recently proposed as a novel neuroimaging biomarker for the same diagnostic conversion.^13^ Another recent study tested a novel multi-modality neuroimaging model, in which structural MRI was combined with fluorodeoxyglucose positron emission tomography (PET), with promising results showing a good performance when predicting further AD diagnosis in MCI patients.^14^

Additionally, multiple studies have identified CSF biomarkers to be clinically relevant for early diagnosis of dementia, particularly high levels of amyloid-β (Aβ)_42_, Aβ_40_, total tau, and phosphorylated tau.^15–17^ Extracellular amyloid plaques comprising Aβ peptides are regarded as an important neuropathologic feature of AD, with their assessment being typically performed by lumbar puncture or amyloid PET scans. Recent literature has suggested a greater predictive value of the ratio Aβ_42_/Aβ_40_ for predicting early stages of dementia and AD, in comparison with other CSF biomarkers.^17,18^ Positive plasma Aβ_42_/Aβ_40_ has been highlighted as a potential independent predictor of current and further brain amyloidosis and for diagnosis conversion to AD, even when amyloid PET is negative.^15^,^16^

The available literature has highlighted the importance of measuring cognitive disfunction in individuals with MCI, as it is regarded as an important marker to identify early stages of dementia, including AD.^1,2,5,6^ Recent longitudinal studies have confirmed the predictive value of cognitive assessment for the early detection of AD,^19–21^ although the available evidence is still limited. Common instruments that have been used to assess cognitive disfunction in MCI patients include the Mini-Mental State Examination (MMSE),^22^ the Alzheimer's Disease Assessment Scale—Cognitive Subscale (ADAS-Cog),^23^ and the Clinical Dementia Rating scale Sum of Boxes (CDR-SB).^24^ Previous research suggests that scores from ADAS-Cog and CDR-SB might offer greater assessment precision for severity of cognitive dysfunction in individuals with different cognitive profiles (healthy, MCI and AD).^25^

The comorbidity of AD and depression is well-know, although the nature of this association remains unclear.^26^ The literature is ambiguous on whether depression predicts diagnostic conversion from MCI to AD, and whether it is regarded as a prodrome to AD.^26^ Nevertheless, there is a growing body of literature showing that depression itself can be a risk factor for further AD and other forms of dementia, even when depression occurs years before the onset of AD.^27^ The literature has highlighted that depression associated with AD can also occur as a reaction to the disease and its related difficulties.^28^ The evidence of pathological mechanisms underlying the link between depression and AD is still preliminary and mostly based on pre-clinical research, with the literature drawing attention to hypothalamic-pituitary-adrenal (HPA) dysregulation, neuroinflammation, and deficits in the 5-HT receptors.^26^

Other potential sociodemographic and clinical factors of diagnosis conversion from MCI to AD have been identified. According to a large cohort study using a novel machine learning-based model, factors such as the age at onset of MCI, ongoing obstructive sleep apnea, long-term opioids use, spondylosis, brain damage, and viral infection diseases of the abdomen (e.g., hepatitis C), can have an interesting clinical predictive value for the progression from MCI to AD.^4^

In this article, we want to contribute to the investigation of longitudinal predictors of diagnostic conversion from MCI to AD, using contemporary and robust advanced statistical methods for predictive modelling, particularly Bayesian hierarchical models, which have been regarded as powerful statistical tools in clinical research.^29,30^ Previous research has highlighted the high predictive value of neuroimaging biomarkers to detect diagnostic conversion to AD, but evidence is still limited on the actual predictive value of other non-invasive clinical measures such as cognitive and neuropsychiatric assessments. These assessments might offer a less expensive and faster route to screen patients for early detection of AD. Better evidence on predictors of further diagnostic conversion to AD is of paramount importance, as it can contribute to better clinical decision making when managing early stages of dementia and choosing patient best treatment options.

## Methods

### Study design

The current research is an observational retrospective longitudinal study of patient data from the Alzheimer's Disease Neuroimaging Initiative (ADNI) database (adni.loni.usc.edu).^31^ ADNI is a longitudinal multi-center observational study, whose main goal is to validate biomarkers for AD clinical trials. The ADNI study addresses disease progression using follow-up assessments of biological markers, including neuroimaging and CSF biomarkers, and cognitive function assessed by cognitive and neuropsychological tests. In the ADNI study, patient follow-up assessments comprise periodic visits, in which patients can be assessed for cognitive function, blood biomarkers, CSF, changes in the brain structures using MRI and PET scans, and psychopathological symptoms. At each patient visit a diagnosis is made by clinicians, based on available medical data. Patients’ medical history and sociodemographic data is collected at baseline.

In our previous study with the ADNI patient data, the diagnostic conversion from MCI to AD was investigated via single-modality and multi-modality neuroimaging classification approaches using machine-learning based models.^14^ Findings included a novel multi-modality approach combining structural MRI with fluorodeoxyglucose positron emission tomography, with a classification performance (balance accuracy) for diagnostic conversion of 78.4%. The current study intends to continue previous work by examining the potential classification performance of other clinical variables such as cognitive function, psychopathological symptoms, CSF biomarkers, and socio-demographic patient variables (age at baseline, sex, education), as predictors of diagnostic conversion from MCI to AD. The current study is focused on examining cases of first-time diagnosis of AD, i.e., no cases of diagnostic reconversion (from AD to MCI) were considered.

### Participants

For the current study, a sample of 572 patients with valid data at baseline medical visit was extracted from the ADNI patient data repository. Participants from ADNI-1 to ADNI-3 and ADNI GO were eligible for this study. Eligibility criteria included: diagnosis of MCI at baseline; have completed cognitive, CSF, psychopathological, and sociodemographic assessments at baseline; and follow-up medical assessments, including a valid diagnosis (MCI or AD). At the time data was collected (June 2024) we identified patient assessment data for a total follow-up of 198 months from baseline, with multiple intermediate follow-up assessments included. Full patient data for all follow-up assessments as extracted from ADNI data repository is presented in the Supplemental Material (see Supplemental Figure 1). For the current study, two subsampled cohorts were extracted from the original ADNI patient data, to include 36-month and 48-month medical follow-up assessments (details in Data Preprocessing section).

### Measures

The study outcome measure is the formal medical diagnosis, MCI or AD, which is made at the medical visit during the medical follow-up. Independent variables comprised measures of cognitive function / decline, psychopathological symptoms, CSF biomarkers, and patient socio-demographical data.

Measures for cognitive function/decline include the MMSE^22^ and CDR-SB.^24^ The MMSE and the CDR-SB are widely used standardized instruments to assess cognitive impairment for staging dementia due to Alzheimer's disease.^6,25^

The assessment of psychopathological symptoms included the Geriatric Depression Scale (GDS),^32^ and the Neuropsychiatric Inventory Questionnaire (NPIQ).^33^ The GDS is a standardized questionnaire to assess symptoms of depression in older adults. The instrument has been validated for different clinical settings, including in inpatients and outpatients with mild to moderate cognitive impairment.^34^ The NPIQ assesses neuropsychiatric symptoms and psychopathology, including depression, anxiety, apathy, sleep disturbances, changes in appetite, disinhibition, irritability, delusions, hallucinations, agitation, euphoria, and aberrant motor behaviors. The instrument has been validated and widely used in patients with neurological disorders.^35^

CSF biomarkers (obtained via lumbar puncture) were assessed using the ratio Aβ_42_/Aβ_40_ calculated for each medical visit (when available). Patient socio-demographical data collected at baseline included age at baseline, sex, and education.

### Data preprocessing and statistical analysis

All data preprocessing and statistical analyses were performed using *R-Studio* (version 2024.12.0).^36^ Data preprocessing included the following procedures (in the same order): 1) to subsample data extracted from the ADNI repository to maximize number of observations across our study variables and throughout the medical visits. Data was exclusively subsampled for medical visits, to include (for the main cohort) medical assessments at baseline, 12, 24, and 36 months; 2) to remove participants with missing data for the full medical follow-up (baseline to month 36), in any variable included (medical assessments or patient demographical data); 3) to standardize all continuous independent variables using the proportion of maximum scaling method (POMS), a specific normalization method for longitudinal data,^37^ in which (x(observation)−min(x))/(min(x)−max(x)) . This method allows us to avoid bias coming from other standardization methods such as z-scores obtained from datasets containing between and within-subject data^37^; and 4) to run multiple imputation procedures to input missing values in our datasets.

The main subsampled cohort (baseline to 36 months) allowed us to include the greatest number of observations for all cognitive, neuropsychiatric and demographical variables throughout the medical visits. The variable CSF biomarkers (ratio Aβ_42_/Aβ_40_) was not included in the main cohort due to its high proportion of missing cases throughout the 36-month medical follow-up. In the primary data extracted from ADNI, most patients had available CSF biomarkers (ratio Aβ_42_/Aβ_40_) assessments at baseline and months 24 and 48, with a considerably reduced number CSF assessments for other medical visits (see Supplemental Figure 1). For this reason, two separate cohorts were subsampled from the original dataset extracted from the ADNI repository: Cohort 1 (main): patient data for medical visits at baseline, plus follow-up assessments at months 12, 24 and 36. This dataset contained all demographical variables plus cognitive (CDR-SB; MMSE) and neuropsychiatric measures (NPIQ; GDS); Cohort 2: patient data for medical visits at baseline, plus follow-up assessments at month 24 and 48. This dataset included all demographical, cognitive and neuropsychiatric measures plus the variable CSF biomarkers (ratio Aβ_42_/Aβ_40_). Multiple imputation was performed for both datasets, assuming that missing observations were at random, using fully conditional specification implemented through the *MICE* algorithm, which adopts multivariate imputation by chained equations, using the *mice* R package.^38^ 10 dataset imputations were used for all variables with missing values. The percentage of missing values for the variables included ranged from 0.7% to 18.7% in cohort 1, and from 4.7% to 47.4% in cohort 2 (full details on missing data for each dataset presented in the Supplemental Material).

**Figure 1.**
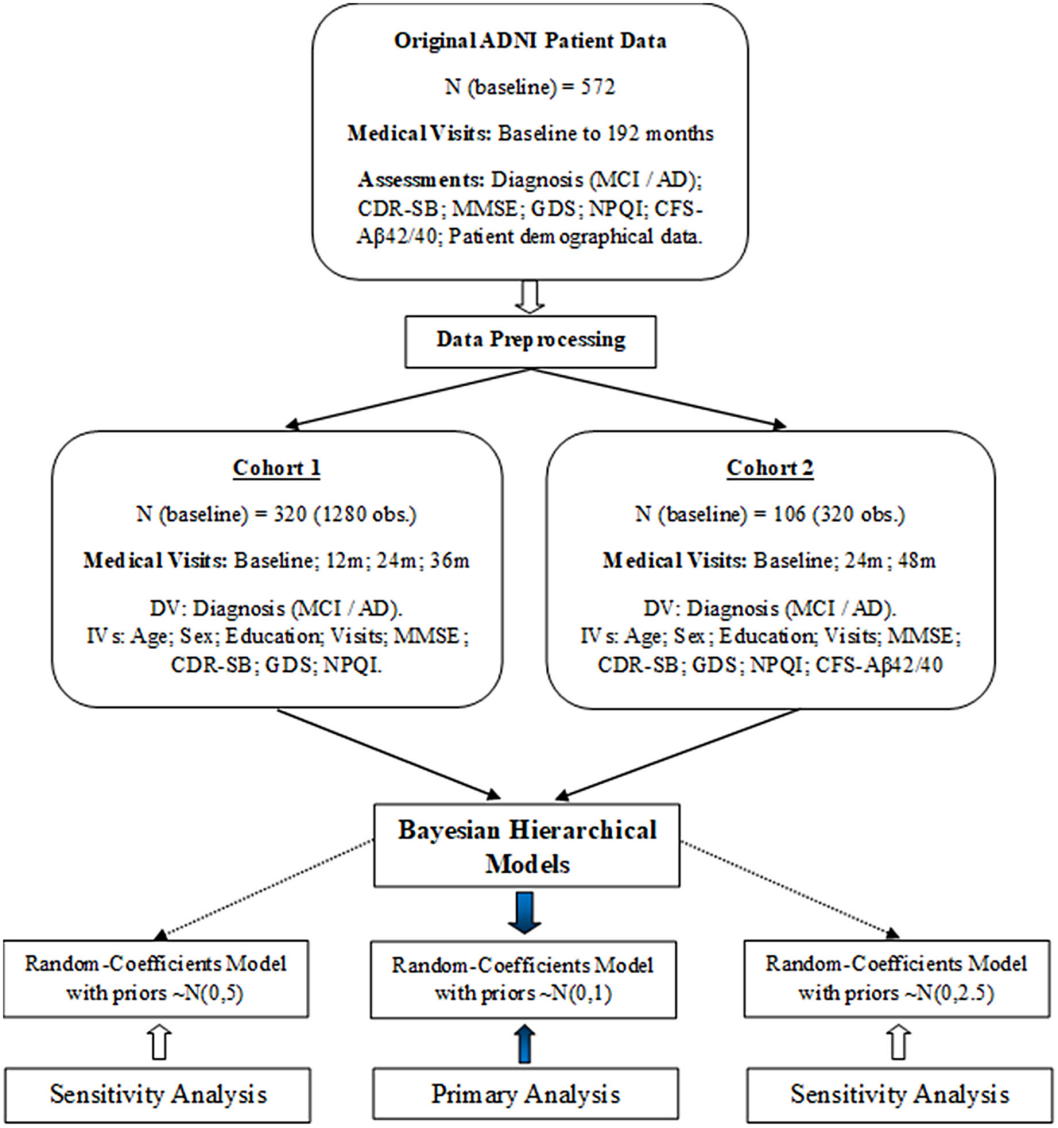
Data preprocessing and statistical analysis strategy.

The full data analysis strategy is presented in Figure 1. Bayesian hierarchical models were computed to investigate predictors of diagnostic conversion from MCI to AD during the medical follow-up included in our two cohorts. Unlike the frequentist statistical approach, Bayesian estimation models allow us to quantify uncertainty based on prior information on the distributions of parameters of interest. Bayesian estimation has become popular in medical research,^29,30,39^ and is regarded as a powerful statistical method for handling relatively small samples entailing clustered or multilevel data at higher precision, in comparison with the frequentist statistical approach.^40,41^ In addition, Bayesian hierarchical models account for both fixed and random effects. This can be advantageous in longitudinal data containing individual trajectories for which a random effect is expected to be associated with.

Bayesian binomial generalized mixed-models were performed considering the longitudinal design of this study and the binary outcome measure (diagnosis: MCI or AD). Random-coefficients models were adopted as they allow us to model intra-individual variability for the intercept and for the slopes (individual trajectories over time), plus the fixed-effects and corresponding interactions.

In Bayesian estimation, informative priors are generally preferred over non-informative priors as they incorporate prior evidence, thus improving estimation precision.^40,42,43^ However, when reliable prior knowledge is lacking, as is the case for the predictive value of cognitive and neuropsychiatric measures in MCI-to-AD conversion, informative priors may introduce bias. At the same time, recent literature has shown that non-informative (flat) priors can lead to overestimated effect sizes, contradicting their supposedly neutral role.^42–46^ In light of this, weakly-informative priors have been recommended as a suitable alternative in contexts with limited prior knowledge, offering guidance to estimation without introducing substantial bias.^44,45^ For the main analysis, we adopted a weakly-informative prior of N(0,1) for fixed effects (β), and a half-normal prior for random effects (τ). Two additional models incorporating slightly vaguer weakly-informative priors were used in a sensitivity analysis. This strategy aligns with current Bayesian literature, especially in generalized models with limited prior evidence, where flat or overly vague priors (e.g., N(0100)) are generally discouraged.^45–47^^[Bibr bibr46-13872877251360228]^

## Bayesian generalized mixed-model (random-coefficients):

The outcome, diagnosis (MCI or AD), is modelled using a Binomial distribution

***Diagnosis_it_* ∼ *Binomial*(** $\gamma_{it}$ **)**

$$logit(\gamma_{it})=\beta_{00}+\beta_{1}X1_{i}+\beta_{2}X2_{i}+\beta_{3}X3_{i}+\beta_{4}X4_{it}+\beta_{5}X5_{it}+\beta_{6}X6_{it}+\beta_{7}X7_{it}+\beta_{8}X4_{ij}X5_{it}+\beta_{9}X4_{ij}X6_{it}+\beta_{10}X4_{ij}X7_{it}\;+\tau_{0t}+\tau_{it}+R_{it}$$

Model for the response binary variable γ (MCI or AD), with the covariates age at baseline, (X1), sex (X2), education (X3), assessment visit (X4), CDR-SB (X5), GDS (X6), CSF (Aβ_42_/Aβ_40_) (X7) and the temporal interactions X4:X5, X4:X6, and X4:X7. i = 1…n individuals, and t = 1 to 2 or 3 assessment visits (time). β represents the fixed-effects (level 1) and τ_0t_ and τ_it_ represents the random-effects at individual level (intercept) and for the individual slopes respectively. Residuals are represented by R_it_.

### Model prior distributions:

**Model 1** (main): β_00,_ β_1,_ β_2_, β_3_, β_4_, β_5_, β_6_, β_7,_ β_8_, β_9,_ β_10_ : ∼ N(0,1); τ_0i,_ τ_it_: ∼ half-N(0,1) *##Wweakly-informative priors*
**Model 2** (sensitivity analysis): β_00,_ β_1,_ β_2_, β_3_, β_4_, β_5_, β_6_, β_7,_ β_8_, β_9,_ β_10_ : ∼ N(0,2.5); τ_0i,_ τ_it_: ∼ half-N(0,2.5) *##Alternative weakly-informative priors.*
**Model 3** (sensitivity analysis)**:** β_00,_ β_1,_ β_2_, β_3_, β_4_, β_5_, β_6_, β_7,_ β_8_, β_9,_ β_10_ : ∼ N(0,5); τ_0i,_ τ_it_: ∼ half-N(0,5)*## Alternative weakly-informative priors.*

All Bayesian models were run with the *brms* R package which uses the Stan probabilistic programming language and implements the No-U-Turn Sampler (NUTS) extension of the Hamiltonian Monte Carlo algorithm.^48,49^ Models were set with 4000 iterations, 2000 warm-up, 4 chains, 4 cores, using within-chain parallelization. The diagnostics (statistical assumptions) for Bayesian models included the inspection of R-hat values, trace plots, posterior predictive distribution plots, ppcheck plots, cummulative quantile plot, and effective sample sizes.

## Results

**Figure 2.**
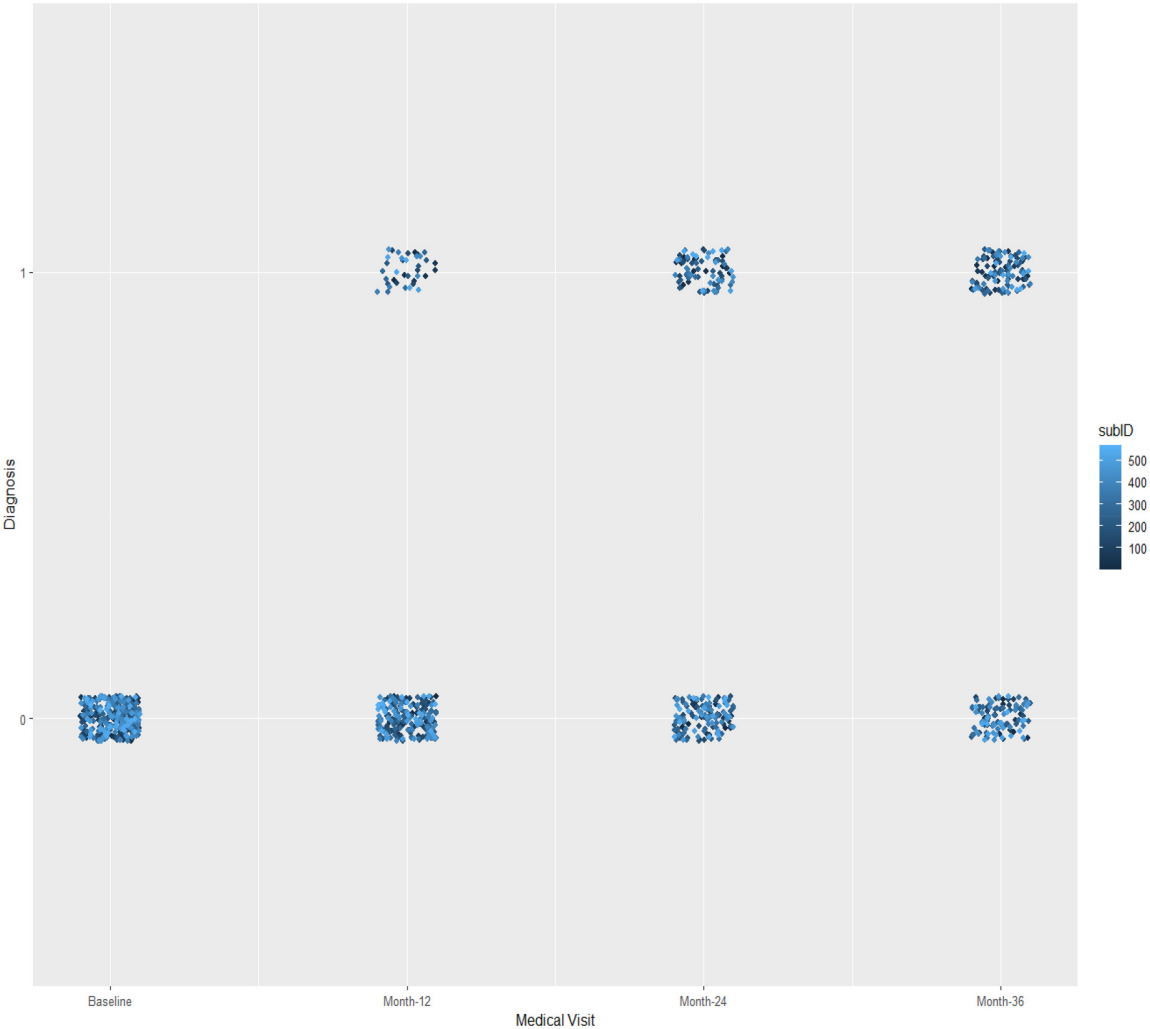
Distribution of the dependent Variable “diagnostic” throughout the medical visits. Variable “Diagnostic”: 0 = Mild Cognitive Impairment; 1 = Alzheimer's Disease; subID: subjects (total observations.

Participants’ characteristics at baseline are presented in Table 1. The distribution of the dependent variable “Diagnostic” throughout the 36-month follow-up is presented in Figure 2.

**Table 1.** Sample characterization at baseline.

	Primary Dataset Extracted from the ADNI Data Repository (N = 572)			Datasets used for Analysis			
				Cohort 1* (N = 320)		Cohort 2* (N = 106)	
		N	%	N	%	N	%
Sex	Female	228	39.9%	118	36.9%	41	38.7%
	Male	344	60.1%	202	63.1%	65	61.3%
Education	Up to 9 years	8	1.4%	8	2.5%	0	0%
	10 to 12 years	88	15.4%	50	15.6%	17	16%
	>12 years	476	83.2%	262	81.9%	89	84%

	M (SD)	Min-Max	M (SD)	Min-Max	M (SD)	Min-Max
Age	73.3 (7.3)	55–90	74.4 (7.1)	55–89	72.8 (6.6)	56–88
CDR-SB	1.5 (0.9)	0.5–6.0	1.5 (0.8)	0.5–5	1.3 (0.7)	0.5–3.5
MMSE	27.6 (1.8)	19–30	27.4 (1.8)	23–30	27.8 (1.7)	23–30
NPIQ	2.0 (3.0)	0–24	2.0 (3.0)	0–24	2.2 (3.1)	0–24
GDS	1.7 (1.4)	0–6	1.6 (1.4)	0–6	1.6 (1.4)	0–6
Aβ_42/40_ Ratio	0.1 (0.1)	0.02–0.34	—	—	0.1 (0.1)	0.03–0.3
Medical Visits	From baseline to month 192		Baseline + Months 12, 24, and 36		Baseline + months 24 and 48	

*Cohorts subsampled from the primary ADNI dataset, with non-imputed and non-standardized baseline data; M: Mean; SD: Standard Deviation; Min-Max: sample minimum and maximum. CDR-SB: Clinical Dementia Rating scale—sum of boxes; MMSE: Mini-Mental State Examination; NIPQ: Neuropsychiatric Inventory Questionnaire; GDS: Geriatric Depression Scale.

### Predictors of diagnostic conversion—Bayesian hierarchical models

The results of Bayesian generalized mixed-models (random-coefficients) using different prior distributions are presented in Tables 2 and 3 for cohorts 1 and 2, respectively. All algorithms used in Bayesian models estimation converged, with all R-hat values (Gelman-Rubin convergence statistic for MCMC diagnostics) equal to 1 (Tables 2 and 3). Bayesian model 1 diagnostics, including trace-plots, posterior predictive distributions, posterior check plot, cumulative quantile plot, and effective sample sizes are presented in the Supplemental Material and confirmed model convergence and reliability of posterior estimates.

**Table 2.** Bayesian generalized mixed models with the cohort 1 (random-coefficients).

	MODEL 1 Priors ∼N(0,1)					MODEL 2 Priors ∼N(0,2.5)					MODEL 3 Priors ∼N(0,5)				
	*β*					*β*					*β*				
	*M*	*SD*	*Med*	*HDI (95%)*	*R-hat*	*M*	*SD*	*Med*	*HDI (95%)*	*R-hat*	*M*	*SD*	*Med*	*HDI (95%)*	*R-hat*
*Fixed-effects*															
Age at baseline	−0.1	0.8	−0.1	[−1.58, 1.39]	1.00	−0.6	1.5	−0.6	[−3.51, 2.21]	1.00	−1.4	2.3	−1.4	[−6.10, 3.00]	1.00
Education	−0.4	0.8	−0.4	[−1.83, 1.09]	1.00	−1.0	1.4	−1.0	[−3.81, 1.85]	1.00	−1.8	2.3	−1.8	[−6.10, 2.81]	1.00
Sex (F)	−0.2	0.4	−0.2	[−1.11, 0.62]	1.00	−0.4	0.7	−0.4	[−1.83, 1.04]	1.00	−0.6	1.1	−0.6	[−2.78, 1.52]	1.00
Visits	**1**.**7**	**0**.**5**	**1**.**7**	**[0.77, 2.73]**	1.00	**2**.**1**	**1**.**0**	**2**.**1**	**[0.10, 4.11]**	1.00	2.5	1.8	2.5	[−0.91, 6.02]	1.00
NIPQ	0.1	0.9	0.1	[−1.67, 1.94]	1.00	−0.3	2.1	−0.3	[−4.49, 3.81]	1.00	−2.1	3.9	−2.1	[−9.84, 5.45]	1.00
CDR-SB	**2**.**2**	**1**.**0**	**2**.**2**	**[0.28, 4.03]**	1.00	4.2	2.3	4.2	[−0.48, 8.58]	1.00	6.3	4.5	6.3	[−2.62, 15.03]	1.00
MMSE	−1.8	0.9	−1.8	[−3.66, 0.01]	1.00	−3.4	2.2	−3.4	[−7.86, 0.90]	1.00	−5.5	4.3	−5.5	[−13.87, 2.97]	1.00
GDS	−0.1	0.9	−0.1	[−1.97, 1.70]	1.00	−0.5	2.2	−0.5	[−4.84, 3.82]	1.00	−1.4	4.1	−1.4	[−9.32, 6.79]	1.00
Visit:NIPQ	0.5	0.5	0.5	[−0.51, 1.56]	1.00	0.7	1.0	0.7	[−1.23, 2.75]	1.00	1.6	1.7	1.6	[−1.77, 4.91]	1.00
Visit:CDR-SB	**5**.**6**	**0**.**7**	**5**.**6**	**[4.28, 6.96]**	1.00	**10**.**3**	**1**.**5**	**10**.**3**	**[7.30, 13.34]**	1.00	**15**.**7**	**3**.**0**	**15**.**7**	**[9.97, 21.53]**	1.00
Visit:MMSE	**−2**.**6**	**0**.**5**	**−2**.**6**	**[−3.62, −1.50]**	1.00	**−4**.**2**	**1**.**2**	**−4**.**2**	**[−6.57, −1.97]**	1.00	**−5**.**9**	**2**.**1**	**−5**.**9**	**[−10.16, −1.83]**	1.00
Visit:GDS	0.4	0.5	0.4	[−0.57, 1.40]	1.00	0.6	1.0	0.6	[−1.31, 2.49]	1.00	0.9	1.7	0.9	[−2.32, 4.17]	1.00
*Random-effects*															
τ_00 subject_	0.4	0.3	0.4	[0.00, 1.05]	1.00	0.9	0.7	0.7	[0.00, 2.10]	1.00	1.4	1.0	1.2	[0.00, 3.33]	1.00
τ_it subject_	**1.1**	**0**.**2**	**1**.**1**	**[0.74, 1.44]**	1.00	1.9	0.3	1.9	[1.28, 2.57]	1.00	**2**.**9**	**0**.**6**	**2**.**8**	**[1.82, 3.98]**	1.00
Conditional Bayes R^2^		0.82 (95% CrI [0.78, 0.86])					0.88 (95% CrI [0.85, 0.91])					0.91 (95% CrI [0.88, 0.94])			
Marginal Bayes R^2^		0.59 (95% CrI [0.53, 0.64])					0.63 (95% CrI [0.58, 0.67])					0.64 (95% CrI [0.59, 0.68])			
N (obs.)	1280*					1280*					1280*				

Bayesian models with the Dataset 1 (baseline + months 12, 24 and 36); All analyses performed with multiple imputation; Model 1: using priors β ∼ N(0,1), τ_0i,_ τ_it_: ∼ half-N(0,1); Model 2: using priors β ∼ N(0,2.5), τ_0i,_ τ_it_: ∼ half-N(0,2.5); Model 3: using priors β ∼ N(0,5); τ_0i,_ τ_it_: ∼ half-N(0,5); M: Mean; Med: Median; HPD: *High Density Interval*; SD: Standard Deviation; CDR-SB: Clinical Dementia Rating scale—sum of boxes; MMSE: Mini-Mental State Examination; NIPQ: Neuropsychiatric Inventory Questionnaire; GDS: Geriatric Depression Scale; obs: Number of observations. **In bold** fixed and random-effects with HDI not containing zero. *320 observations per medical visit. CrI: 95% Credible Intervals. 0.221; 0.241; 0.263.

**Table 3.** Bayesian generalized mixed models with the cohort 2 (random-coefficients).

	MODEL 1 Priors ∼N(0,1)					MODEL 2 Priors ∼N(0,2.5)					MODEL 3 Priors ∼N(0,5)				
	* β*					*β*					*β*				
	*M*	*SD*	*Med*	*HDI (95%)*	*R-hat*	*M*	*SD*	*Med*	*HDI (95%)*	*R-hat*	*M*	*SD*	*Med*	*HDI (95%)*	*R-hat*
*Fixed-effects*															
Age at baseline	−0.5	0.8	−0.5	[−1.97, 1.11]	1.00	−1.2	1.6	−1.2	[−4.39, 1.97]	1.00	−2.4	2.9	−2.4	[−8.01, 3.26]	1.00
Education	0.5	0.7	0.5	[−0.91, 1.99]	1.00	1.2	1.5	1.2	[−1.66, 4.17]	1.00	2.3	2.6	2.3	[−2.84, 7.45]	1.00
Sex (F)	−0.6	0.5	−0.6	[−1.67, 0.45]	1.00	−0.9	1.0	−0.9	[−2.96, 0.97]	1.00	−1.4	1.7	−1.4	[−4.79, 1.96]	1.00
Visits	**2**.**0**	**0**.**6**	**2**.**0**	**[0.88, 3.08]**	1.00	**3**.**0**	**1**.**2**	**3**.**0**	**[0.53, 5.39]**	1.00	4.5	2.4	4.5	[−0.17, 9.16]	1.00
Aβ_42_/Aβ_40_	−0.3	0.9	−0.3	[−2.14, 1.51]	1.00	−0.2	2.2	−0.2	[−4.52, 4.16]	1.00	0.2	4.3	0.2	[−8.20, 8.74]	1.00
CDR-SB	1.4	1.0	1.4	[−0.51, 3.32]	1.00	3.0	2.4	3.0	[−1.77, 7.58]	1.00	5.3	4.7	5.3	[−4.14, 14.32]	1.00
MMSE	−1.6	0.9	−1.6	[−3.41, 0.26]	1.00	−2.8	2.3	−2.8	[−7.26, 1.61]	1.00	−4.3	4.4	−4.3	[−13.00, 4.39]	1.00
GDS	−0.1	0.9	−0.1	[−1.88, 1.81]	1.00	−0.4	2.3	−0.4	[−4.79, 4.10]	1.00	−1.2	4.4	−1.2	[−9.79, 7.42]	1.00
NPIQ	0.4	0.9	0.4	[−1.43, 2.19]	1.00	0.8	2.2	0.8	[−3.43, 4.99]	1.00	1.2	4.0	1.2	[−6.76, 8.95]	1.00
Visit:Aβ_42_/Aβ_40_	−0.4	0.7	−0.4	[−1.76, 0.90]	1.00	−0.7	1.5	−0.7	[−3.61, 2.08]	1.00	−1.2	2.7	−1.2	[−6.53, 4.01]	1.00
Visit:CDR-SB	**3**.**2**	**0**.**8**	**3**.**2**	**[1.62, 4.83]**	1.00	**6**.**4**	**1**.**9**	**6**.**4**	**[2.68, 10.06]**	1.00	**11**.**0**	**3**.**7**	**11**.**0**	**[3.92, 18.18]**	1.00
Visit:MMSE	**−2**.**0**	**0**.**6**	**−2**.**0**	**[−3.19, −0.78]**	1.00	**−3**.**7**	**1**.**4**	**−3**.**6**	**[−6.48, −0.93]**	1.00	**−6**.**2**	**2**.**8**	**−6**.**2**	**[−11.77, −0.91]**	1.00
Visit:GDS	0.1	0.6	0.1	[−1.16, 1.32]	1.00	0.2	1.3	0.2	[−2.24, 2.74]	1.00	0.2	2.3	0.2	[−4.34, 4.71]	1.00
Visit:NIPQ	0.7	0.8	0.7	[−0.71, 2.21]	1.00	1.0	1.6	0.9	[−2.07, 4.16]	1.00	1.6	3.0	1.6	[−4.07, 7.43]	1.00
*Random-effects*															
τ_00 subjid_	0.5	0.4	0.4	[0.00, 1.18]	1.00	0.9	0.7	0.9	[ 0.00, 2.28]	1.00	1.6	1.2	1.3	[0.00, 3.87]	1.00
τ_it subjid_	0.4	0.3	0.3	[0.00, 0.82]	1.00	0.9	0.1	0.9	[ 0.00, 1.69]	1.00	**1**.**8**	**0**.**8**	**1**.**7**	**[0.32, 3.31]**	1.00
Conditional Bayes R^2^		0.66 (95% CrI [0.55, 0.77])					0.80 (95% CrI [0.69, 0.88])					0.87 (95% CrI [0.78, 0.94])			
Marginal Bayes R^2^		0.58 (95% CrI [0.48, 0.66])					0.67 (95% CrI [0.57, 0.73])					0.69 (95% CrI [0.59, 0.75])			
N (obs.)	320*					320*					320*				

Bayesian models with the Dataset 2 (baseline + months 12, 24 and 36); All analyses performed with multiple imputation; Model 1: using priors β ∼ N(0,1), τ_0i,_ τ_it_: ∼ half-N(0,1); Model 2: using priors β ∼ N(0,2.5), τ_0i,_ τ_it_: ∼ half-N(0,2.5); Model 3: using priors β ∼ N(0,5); τ_0i,_ τ_it_: ∼ half-N(0,5); M: Mean; Med: Median; HPD: *High Density Interval*; SD: Standard Deviation; CDR-SB: Clinical Dementia Rating scale—sum of boxes; MMSE: Mini-Mental State Examination; NIPQ: Neuropsychiatric Inventory Questionnaire; GDS: Geriatric Depression Scale; obs: Number of observations. **In bold** fixed and random-effects with HDI not containing zero. *106 observations per medical visit.

**Figure 3.**
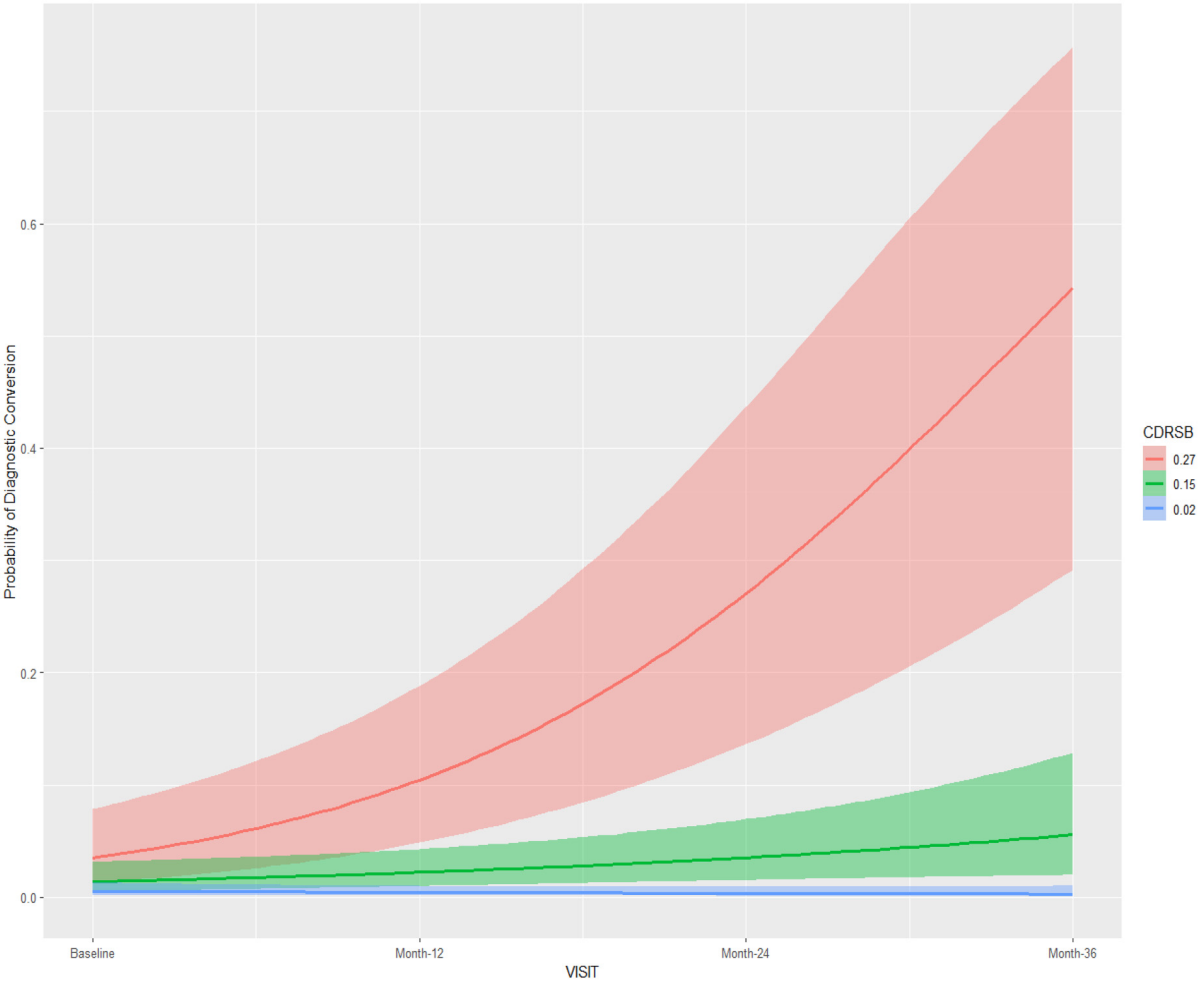
Posterior conditional effects for the interaction visit:CDR-SB on the diagnostic conversion from mild cognitive impairment to Alzheimer's disease. CDRSB: Cognitive Dementia Rating Scale; Standardized values: mean ±1 standard deviation.

**Figure 4.**
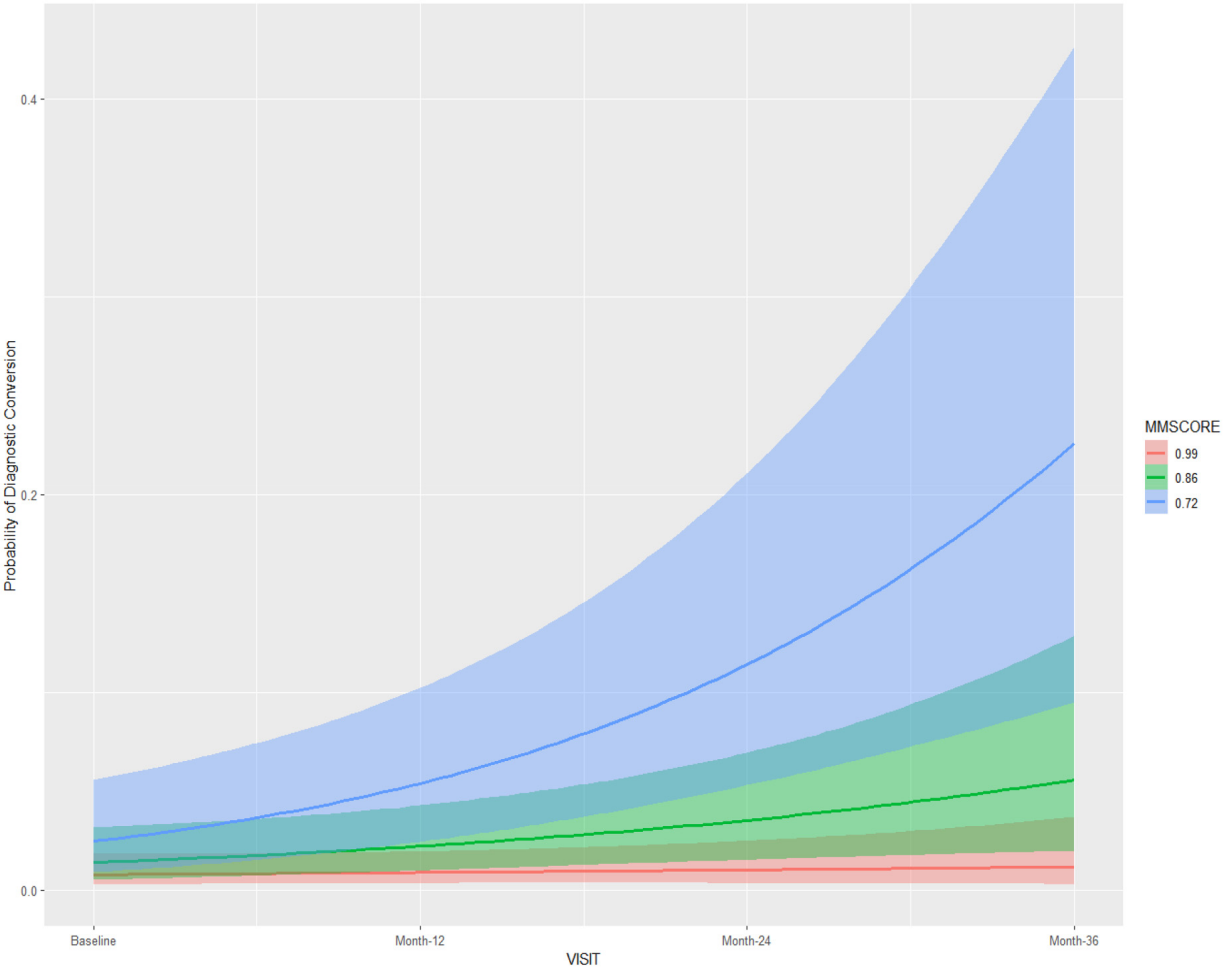
Posterior conditional effects for the interaction visit:MMSE on the diagnostic conversion from mild cognitive impairment to Alzheimer's disease. MMSE: Mini-Mental State Examination; Standardized values: mean ±1 standard deviation.

**Figure 5.**
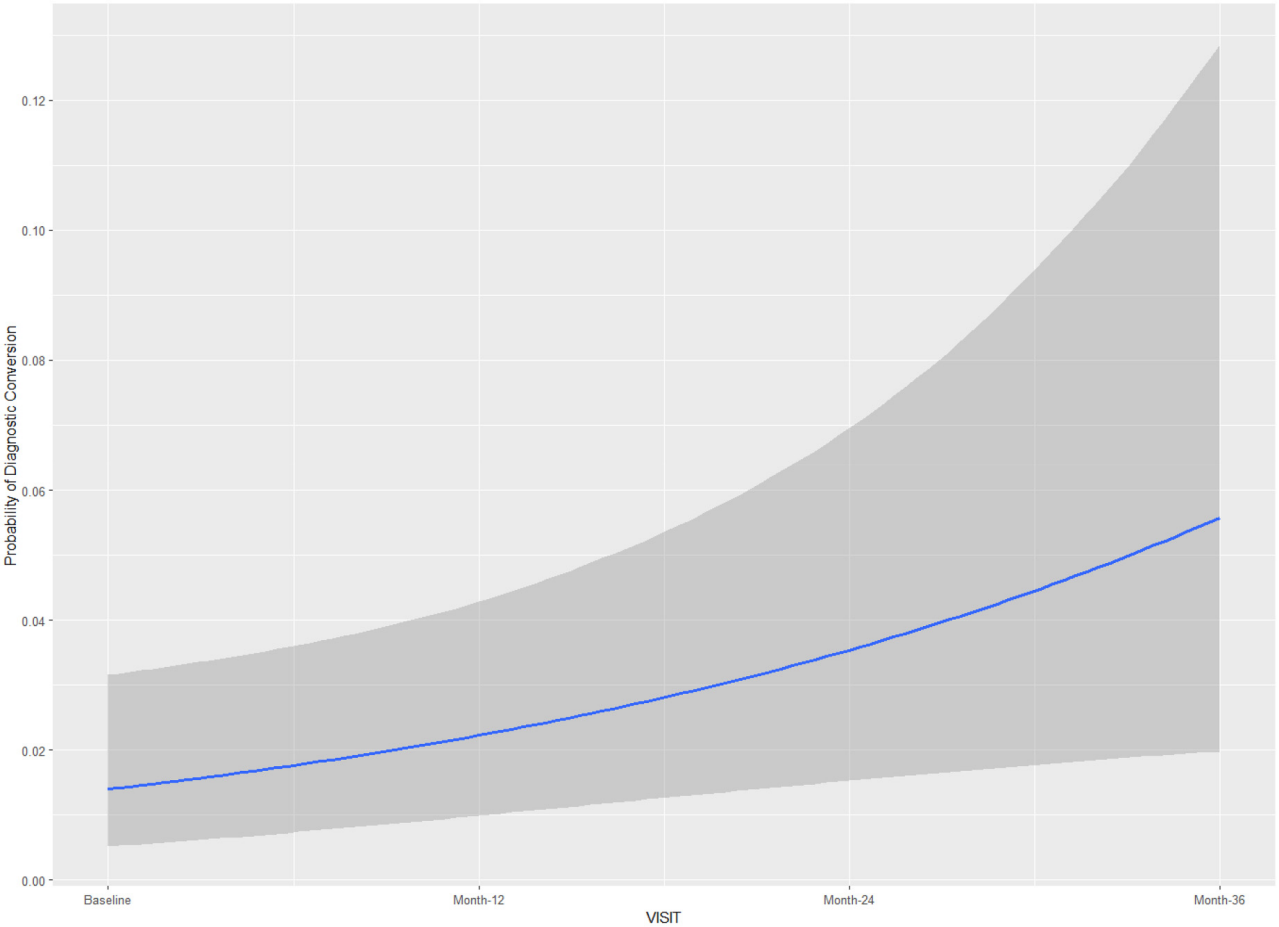
Posterior conditional effects for medical visits on the diagnostic conversion from mild cognitive impairment to Alzheimer's disease.

Results using the cohort 1 (Table 2) suggested the interactions *visit:CDR-SB* and visit:MMSE to have considerable influence on the outcome measure (diagnostic conversion), with the highest density intervals (HDI) for these fixed effects not containing zero in all three models using different prior distributions. The posterior conditional effects for the interactions *visit:CDR-SB and visit:MMSE* in Model 1 (cohort 1) are illustrated in Figures 3 and 4 respectively. Higher scores on CDR-SB were associated with increased risk for a diagnosis conversion, approximately 30% greater probability at 24-month follow-up, and more than 50% probability at 36-month follow-up. Higher scores on MMSE were associated with approximately 10% greater probability of diagnostic conversion to AD at 24-month follow-up, and 20% greater probability at 36-month follow-up. In models 1 and 2, time itself (medical follow-up) was associated with a slightly increased risk for diagnostic conversion to AD, with individuals at 36-month follow-up showing an approximately 5% greater probability of diagnostic conversion to AD (Table 2; Figure 5). The random-effects for individual slopes (τ_it subject_; medical follow-up)) showed an influence on the outcome measure across models with different weakly-informative priors. Conditional and Marginal R^2^ for all models are presented in Table 2. Marginal R^2^ ranged from 0.59 to 0.64, suggesting that 28% to 30% of the model variance (across models using different priors) is due to random-effects.

Results using the cohort 2 were very similar to those found with cohort 1, as the same fixed-effects were suggested to have an effect on diagnostic conversion to AD (Table 3). The CSF biomarker Aβ-42/40 ratio was not found to influence the diagnostic conversion to AD in all models, with the corresponding HDI not containing zero in all models. Random-effects for individual slopes (τ_it subject_; medical follow-up) showed influence on the outcome measure only for model 3 (using vaguer weakly-informative priors). Conditional and Marginal R^2^ for all models are presented in Table 3, suggesting that 12% to 20% of the model variance (across models using different priors) is due to random-effects.

## Discussion

A cornerstone of regular cognitive assessment in older adults with MCI is the early detection of AD, which is key to enhance treatment decision-making for AD patients. The current study contributes to increase the level of evidence regarding the predictive value of cognitive assessment as part of medical follow-up for detecting diagnostic conversion from MCI to AD. Bayesian hierarchical models suggested CDR-SB to be the cognitive assessment tool with the greatest predictive value for early detection of AD over a period of 36 months.

The CDR-SB is a widely validated cognitive measure that provides reliable input on patient cognitive status, which is crucial for medical follow-up of older adults with MCI. The instrument can also capture patient perceptions of cognitive performance, which has been identified as a good predictor of diagnostic conversion from MCI to AD.^50,51^ In particular, previous research has highlighted the potential predictive value of informant-related memory complaints for diagnostic conversion over a period of 4 years.^50^ Our findings are consistent with a previous study suggesting that both CDR-SB and ADAS-Cog perform better for detecting diagnostic conversion from MCI to AD, in comparison with other cognitive measures.^25^ In our study, the MMSE also showed a reliable performance to detect diagnostic conversion to AD over time, although with considerably poorer performance in comparison with the CDR-SB.

In our study, Bayesian hierarchical models suggested the GDS and the NPIQ instruments not to have a predictive value for diagnostic conversion. Previous research is unclear regarding the actual importance of depression and other neuropsychiatric symptomatology as a possible red flag for dementia in older adults with MCI, as well as the nature of the comorbidity between depression and AD.^26,28^ The lack of previous longitudinal studies addressing the role of depression symptomatology as a predictor of diagnostic conversion is one of the main reasons for the lack of evidence in this topic. The available literature from large-scale cohort studies suggests that depression, particularly the severity of depression, is associated with a greater risk for subsequent AD over a period of 20 years.^52^ There is also preliminary evidence suggesting that depression might be a neuropsychiatric symptom of AD, with the onset triggered by the perception of functional limitations caused by the AD.^28,53^ Future larger-scale longitudinal studies should clarify the clinical value of GDS for early detection of AD, and whether the fact that the GDS is a self-report measure can affect its performance as a predictor of diagnostic conversion.

Recent literature has stressed the predictive importance of CSF biomarkers, particularly the CSF Aβ (Aβ_42_/Aβ_40_) ratio for early detection of AD in older adults with MCI.^15–17^ Studies have shown that the Aβ_42_/Aβ_40_ ratio can be the best CSF biomarker to predict diagnostic conversion overtime,^17,18^ together with neuroimaging biomarkers.^12–14^ Our Bayesian hierarchical models did not corroborate the predictive effect of Aβ_42_/Aβ_40_ ratio on diagnostic conversion over a period of 48 months. However, the results might have been affected by considerably high attrition rates over the assessment period (48 months), and the fact that for most patients the Aβ_42_/Aβ_40_ ratio data was only available at baseline, 24 and 48-month medical visits. The Aβ_42_/Aβ_40_ ratio is commonly obtained from lumbar puncture, which is an invasive medical procedure that is not usually done on a regular basis, which might, therefore, explain the limitations in the available Aβ_42_/Aβ_40_ ratio data. Future longitudinal studies with lower attrition rates should clarify the actual predictive value of this variable, as well as its importance in comparison with other predictors of diagnostic conversion.

The use of Bayesian hierarchical models in the context of medical research has proven to be of great value as they accommodate prior information on the parameters of interest and therefore offer more robust and precise predictions while being more flexible for sample size requirements, in comparison with the frequentist approach.^40^ Recent literature has recommended to avoid the use of non-informative or flat prior distributions, in favor of more informative priors, even if they are weakly informative.^42,43,54^ Flat priors have been associated with increased risk of overestimation of the magnitude of effects leading to over-stated evidence on its sign, which in turn contradicts the “non-informative” assumption inherent to these priors.^45^ Furthermore, Bayesian hierarchical models account for fixed and random-effects, which can be advantageous when dealing with longitudinal patient data, whose individual trajectories are of interest. This was corroborated by current results in the main cohort, with a considerable amount of the model variance being due to random-effects, and random-slopes showing a clear predictive effect on the outcome (diagnosis).

Main strengths of the current study include a cohort study design including a reasonable number of patient data collected from different clinical settings, and the use of contemporary and robust statistical methods such as Bayesian hierarchical models. Main study limitations include a considerably high attrition rate in cohort 2, with missing data between the included medical visits which might have limited the statistical models to capture the potential effect of predictors such as the CSF Aβ (Aβ_42_/Aβ_40_) ratio in relation to diagnosis conversion. Also, our predictive models would have strongly benefited from the inclusion of other relevant sociodemographic and clinical variables that have been previously identified as potentially important for diagnostic conversion such as age at onset of MCI, ongoing obstructive sleep apnea, long-term opioids use, spondylosis, brain damage, and hepatitis C. Our study did not include them in as model predictors which is a study limitation to be addressed in future longitudinal research. Finally, the fact that this study only accounted for pre-collected patient data limited our capacity to improve some aspects of the study design that could help reducing attrition rates and data integrity.

In conclusion, the current study highlighted the potential relevance of adopting the CDR-SB as one of the preferred clinical assessment tools in medical follow-up of MCI patients, to help screening for early diagnosis of AD. The use of CDR-SB in MCI patients offers an affordable, fast and non-invasive way to red-flag patients for an early AD diagnosis, which might warrant further medical investigation. Future large-scale cohort studies with lower attrition rates and including multimodal biomarkers will strongly contribute to generate more evidence on which assessment procedures better predict diagnostic conversion and help clinicians to improve care for patients with AD.

## Supplemental Material

sj-docx-1-alz-10.1177_13872877251360228 - Supplemental material for Predicting diagnostic conversion from mild cognitive impairment to Alzheimer's disease: A Bayesian hierarchical model approach using ADNI patient data Supplemental material, sj-docx-1-alz-10.1177_13872877251360228 for Predicting diagnostic conversion from mild cognitive impairment to Alzheimer's disease: A Bayesian hierarchical model approach using ADNI patient data by Hugo Senra, Maria Conceição Costa, Isabel Pereira, Daniel Agostinho, Miguel Castelo-Branco and for the Alzheimer’s Disease Neuroimaging Initiative in Journal of Alzheimer's Disease
