# Supplementary material for: Predicting diagnostic conversion from mild cognitive impairment to Alzheimer's disease: A Bayesian hierarchical model approach using ADNI patient data
Source: J Alzheimers Dis. 2025 Jul 20;107(1):374–87. doi: 10.1177/13872877251360228 (PMC12361702; doi:10.1177/13872877251360228)
Supplement: sj-docx-1-alz-10.1177_13872877251360228 - Supplemental material for Predicting diagnostic conversion from mild cognitive impairment to Alzheimer's disease: A Bayesian hierarchical model approach using ADNI patient data [file sj-docx-1-alz-10.1177_13872877251360228.docx]

**Supplemental Material**

**Predicting diagnostic conversion from mild cognitive impairment to Alzheimer’s disease: A Bayesian hierarchical model approach using ADNI patient data**

**1. Sample Distribution**

**Supplemental Figure 1.** Number of observations per type of medical assessment.

CDR-SB: Clinical Dementia Rating scale – sum of boxes; MMSE: Mini-Mental State Examination; NIPQ: Neuropsychiatric Inventory Questionnaire; GDS: Geriatric Depression Scale; CSF: cerebrospinal fluid.

**Supplemental Figure 2.** Number of cases of diagnostic conversion (Mild Cognitive Impairment to Alzheimer’s Disease) during all medical visits.

**2. Bayesian Hierarchical Models Diagnostics**

**
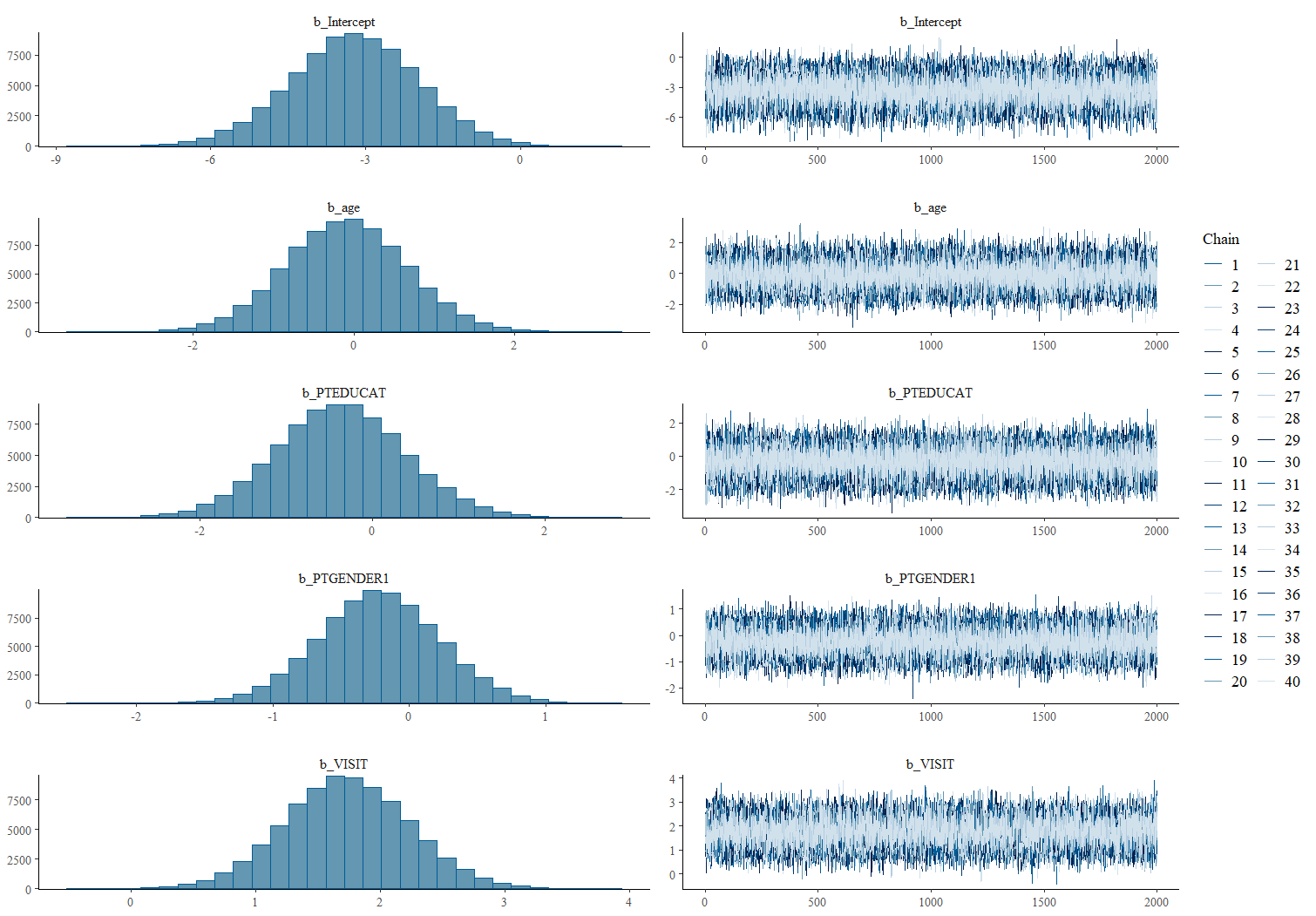
**

**
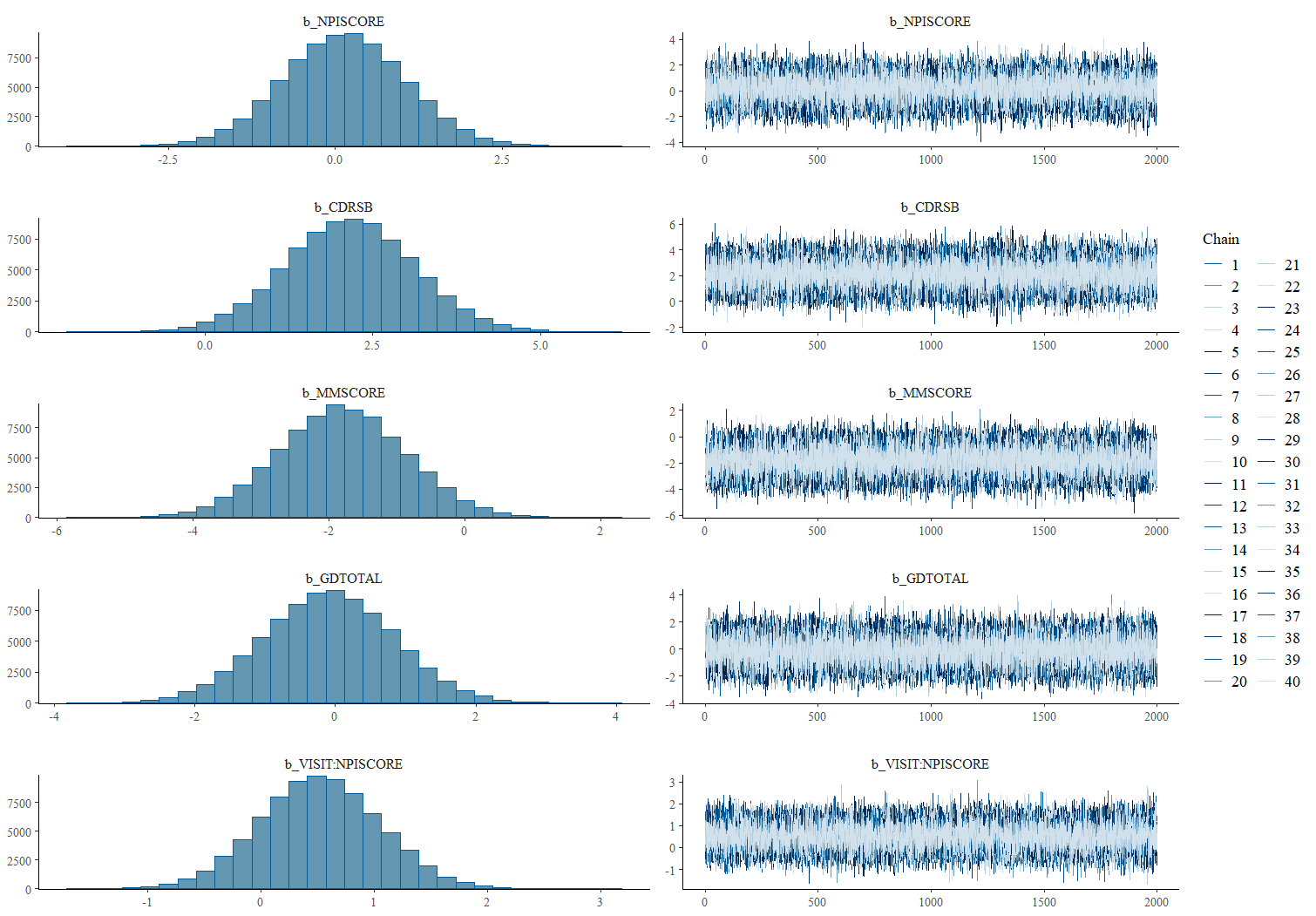
**

**
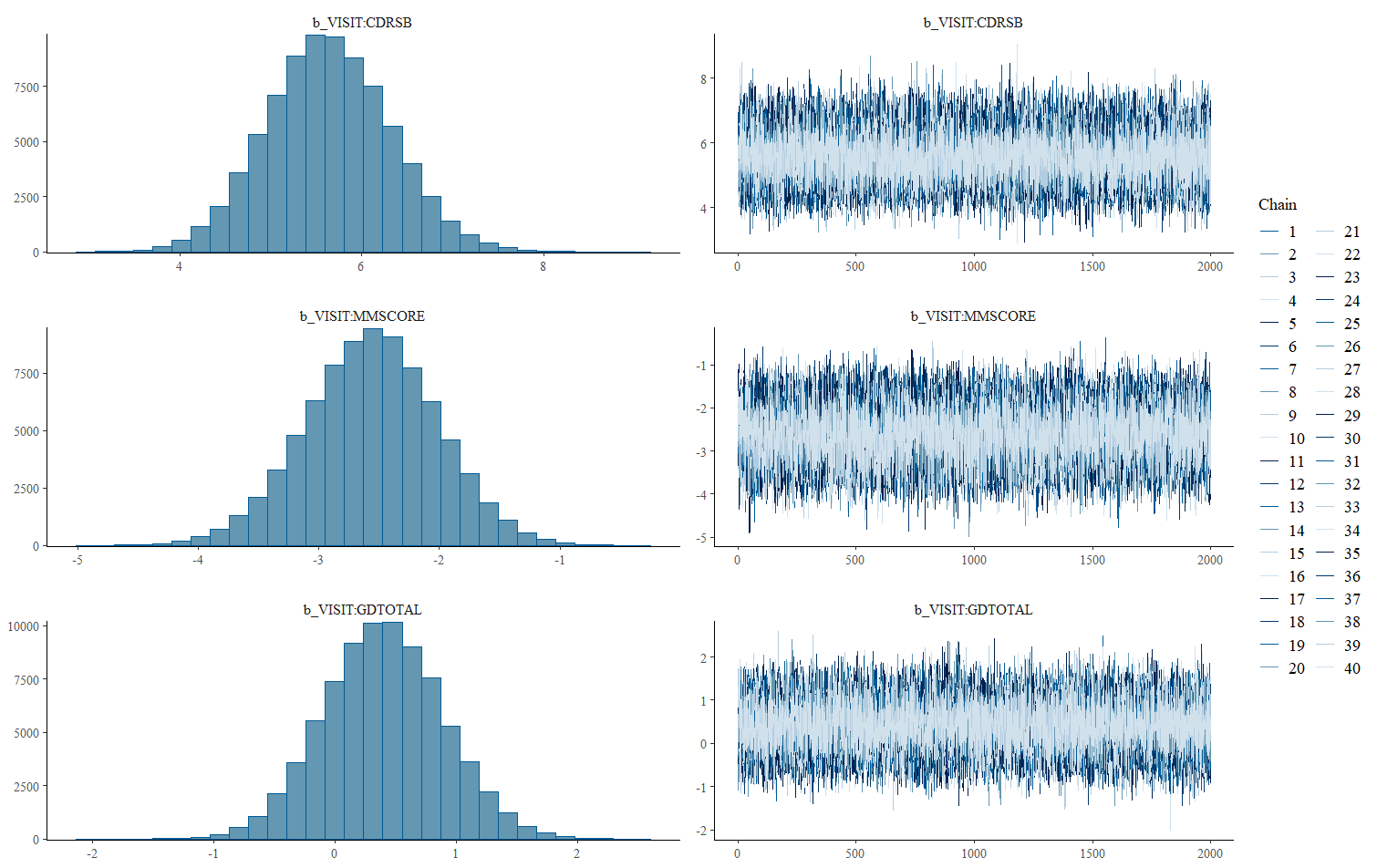
**

**
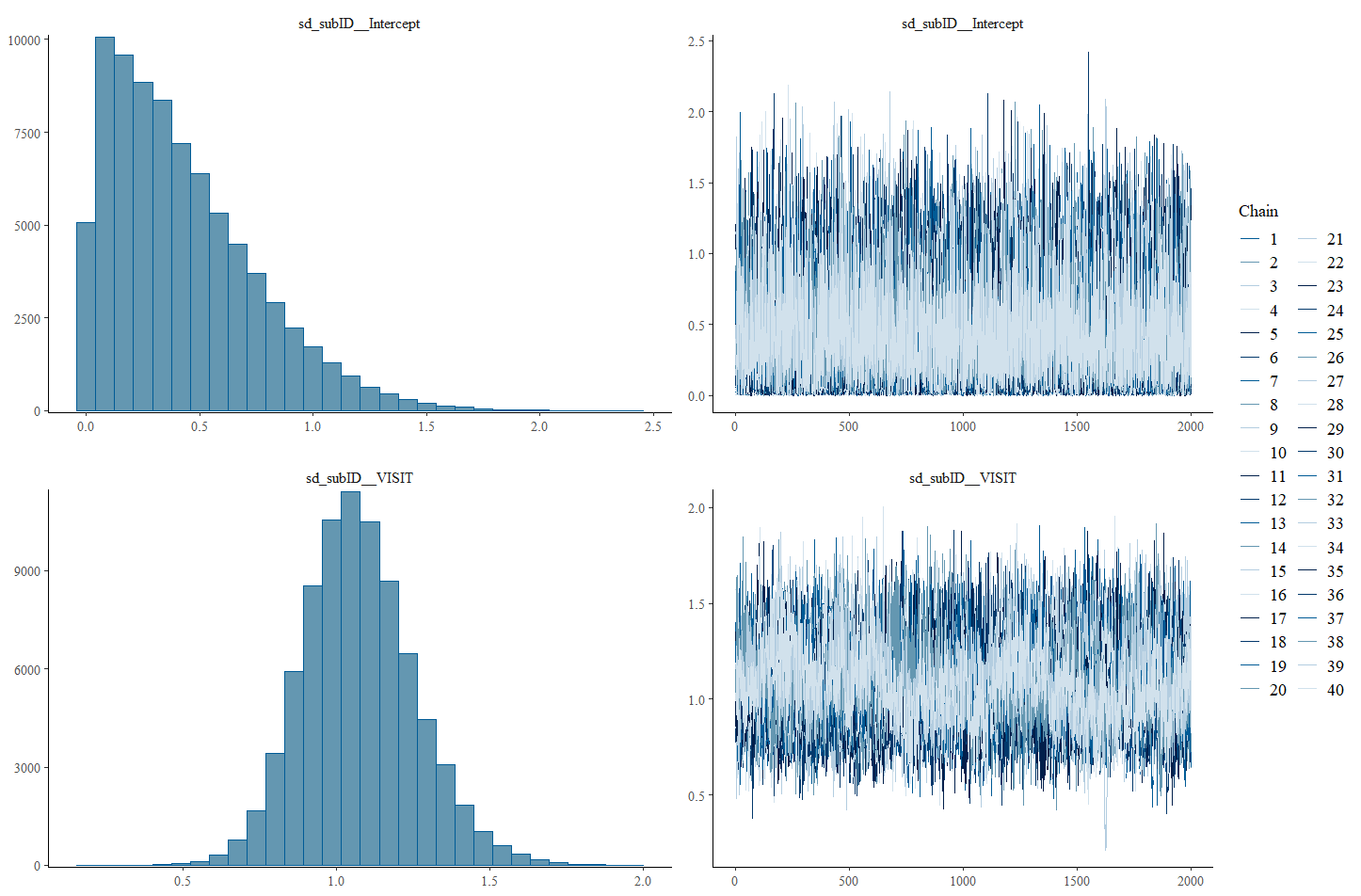
**

**Supplemental Figure 3.** Posterior sample distributions and corresponding trace plots.

**
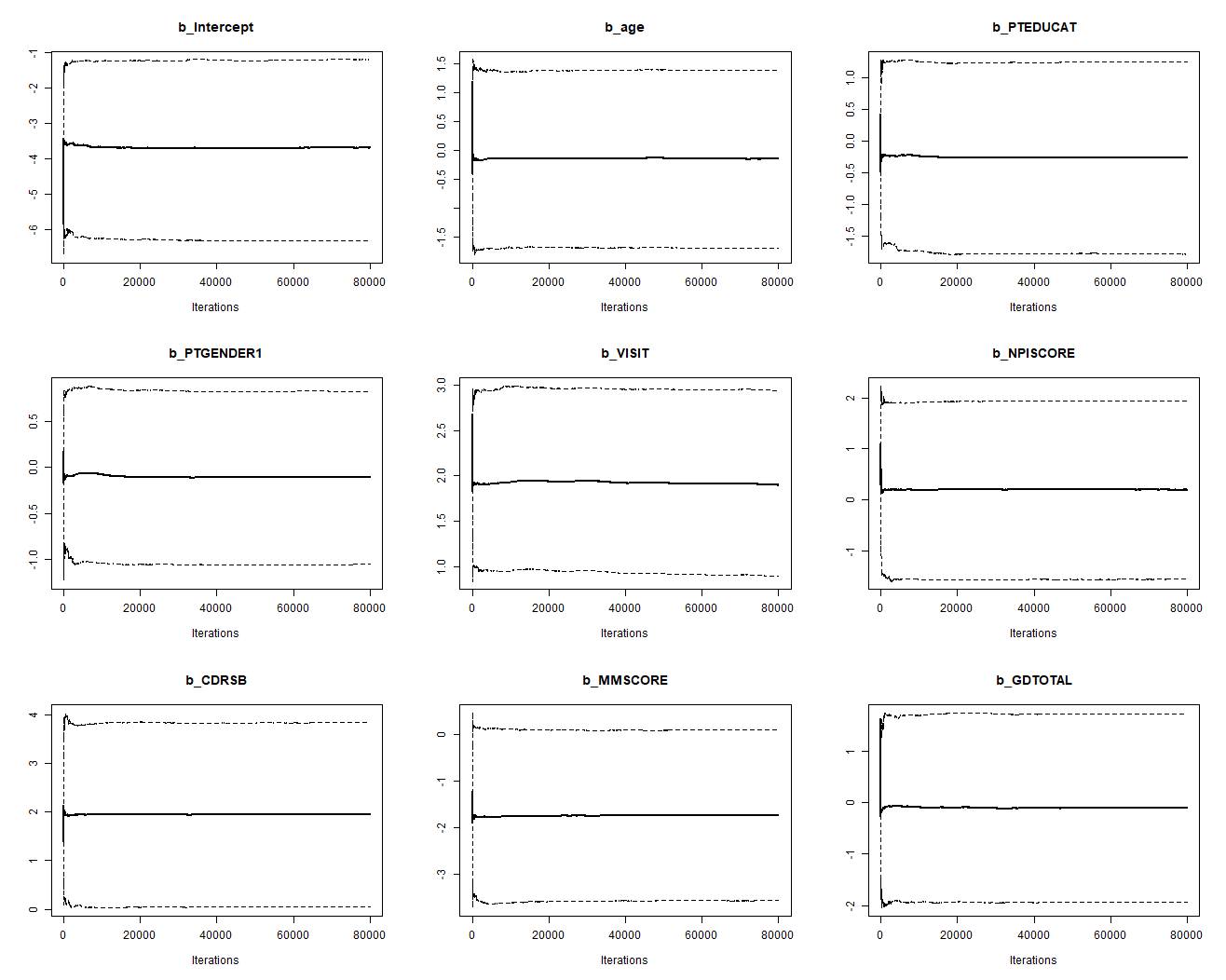
**

**
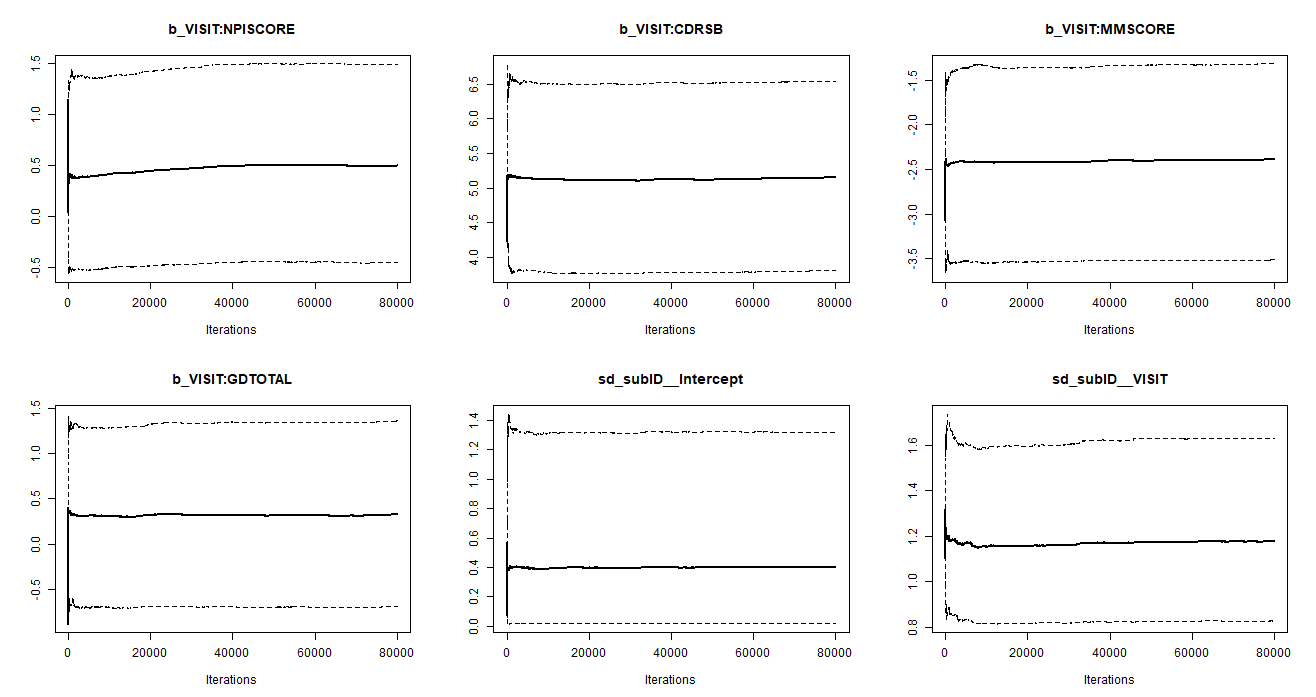
**

**Supplemental Figure 4.** Cumulative Quantile Plot (*cumuplot*)


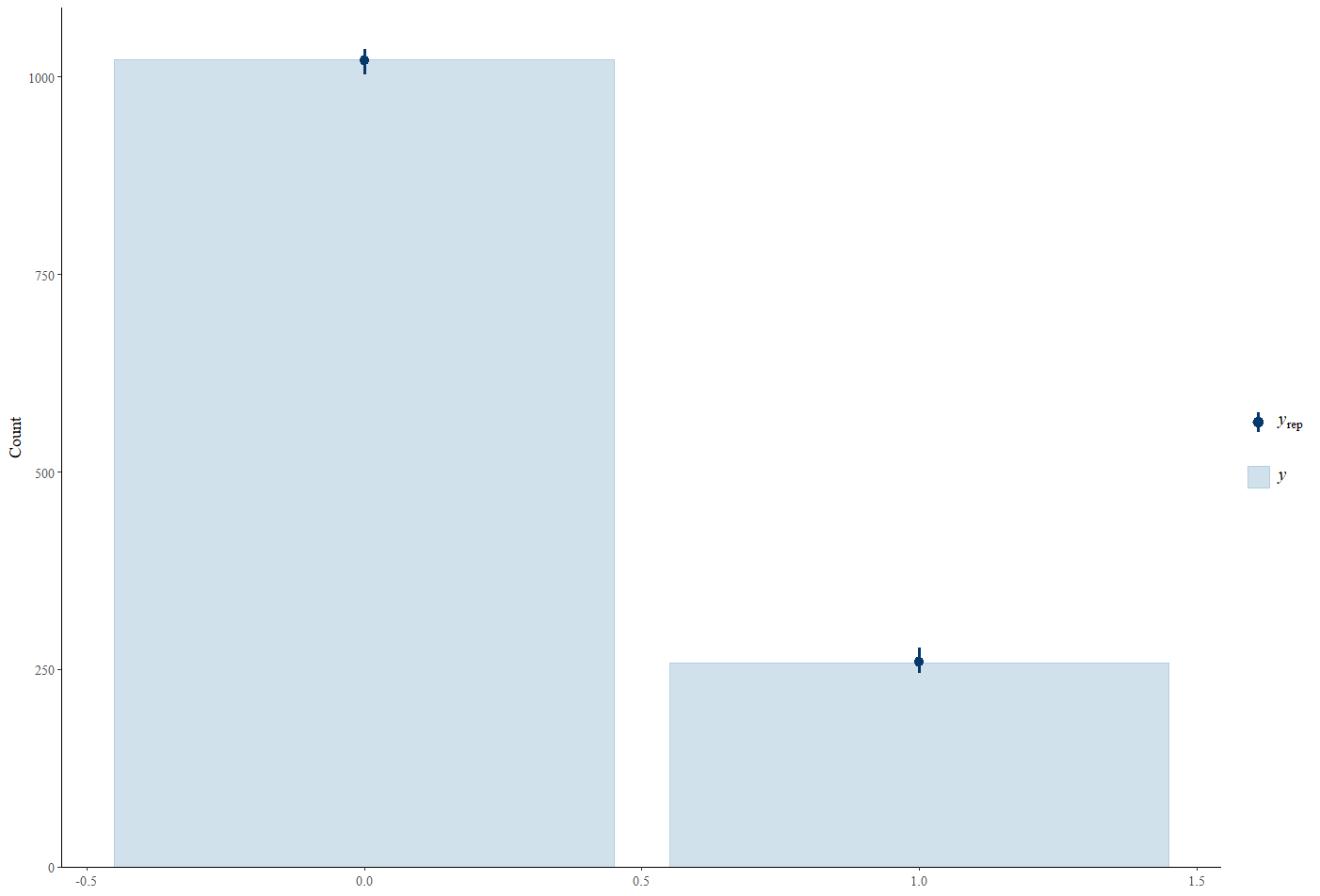


**Supplemental Figure 5.** Posterior predictive checks - ppcheck plot

Binary outcome “Diagnosis” (x-axis), with 0 for “Mild-Cognitive Impairment” and 1 for “Alzheimer’s Disease”
